# Use of mobile devices to answer online surveys: implications for research

**DOI:** 10.1186/1756-0500-6-258

**Published:** 2013-07-08

**Authors:** John A Cunningham, Clayton Neighbors, Nicolas Bertholet, Christian S Hendershot

**Affiliations:** 1 Centre for Mental Health Research, The Australian National University, Canberra, Australia; 2 Centre for Addiction and Mental Health, 33 Russell St, Toronto M5S 2S1, Canada; 3 Department of Psychology, University of Houston, 126 Heyne Bldg, Houston 77204-5022, USA; 4 Alcohol Treatment Center, Department of Community Medicine and Health, Lausanne University Hospital, Lausanne, Switzerland

**Keywords:** Internet, Brief intervention, Alcohol, College, University, Mobile device

## Abstract

**Background:**

There is a growing use of mobile devices to access the Internet. We examined whether participants who used a mobile device to access a brief online survey were quicker to respond to the survey but also, less likely to complete it than participants using a traditional web browser.

**Findings:**

Using data from a recently completed online intervention trial, we found that participants using mobile devices were quicker to access the survey but less likely to complete it compared to participants using a traditional web browser. More concerning, mobile device users were also less likely to respond to a request to complete a six week follow-up survey compared to those using traditional web browsers.

**Conclusions:**

With roughly a third of participants using mobile devices to answer an online survey in this study, the impact of mobile device usage on survey completion rates is a concern.

**Trial registration:**

ClinicalTrials.gov: NCT01521078

## Findings

### Introduction

A common means of recruiting participants for Internet-based interventions trials, particularly in college populations, is to use mass invitations through email lists (e.g., [1-3]). In this procedure, a list of student email addresses is obtained from the college registrar and students are sent an invitation to participate in a research trial that contains a link to a web page which describes the trial and asks a series of questions regarding the behavior under study. The strengths of this method include the speed with which a large sample can be recruited and the ability to proactively engage participants who exhibit the risk behavior under study but who would not normally seek help (e.g., heavy drinking college students).

This method of recruitment has been used fairly extensively over the last decade. But will it remain a profitable way of recruiting participants with the widespread adoption of mobile devices with Internet and email capabilities (e.g., iPhone, Blackberry, Andriod device, iPad)? A Pew Internet Project survey conducted in 2012 found that 66% of US 18–29 year-olds owned cell phones [4]. One hypothesis is that this prevalence of smart phones and other mobile devices could be an advantage to participant recruitment through email invitation because potential participants can access their email quickly from anywhere. An opposing hypothesis has to do with the way things can be displayed on a screen or the ease of answering a survey that has been optimized for a computer screen. This alternate hypothesis posits that mobile devices are a disadvantage because fewer participants who use these devices would complete the survey and/or the online intervention under study. This research note examines both of these hypotheses using results from a recent online intervention trial which employed an email list as its recruitment method.

## Methods

Undergraduates at an American university (N = 10,000) received an email invitation to participate in an online survey of alcohol use [5] in the spring of 2012. The email invitation offered a $5 online gift card in exchange for completing a brief screening survey and included a hyperlink to the web survey. This brief screener served to identify heavy drinkers who would then be enrolled into the intervention trial. Students who accessed the survey were directed to an online consent form. Those who provided informed consent proceeded to the brief screening survey, which consisted only of demographic questions and a 3-item alcohol screener (the AUDIT-C, [6]). The AUDIT-C items assess frequency of drinking, usual quantity of drinking, and number of days on which participants consumed 5 or more drinks (a 3-month reference frame was applied for AUDIT-C questions). Upon completing the survey, participants who met a pre-defined cutoff for heavy drinking (AUDIT-C score of 4 or greater) were asked to agree to a follow-up survey in 6 weeks, whereas those with scores less than 4 were thanked for their participation and screened out of the subsequent trial.

Heavy drinkers who agreed to be contacted for a subsequent survey were randomized to receive immediate access to an online alcohol use intervention program, or to receive no access. Those randomized to the access condition received a prompt to view a website, Check Your Drinking University (CYDU, [7]). The CYDU program, described in more detail elsewhere [5] provides users with brief personalized feedback about their alcohol use, including normative feedback specific to age, sex and geographic region. This web-based feedback program, tailored to undergraduates, is based on a similar screening program shown to reduce alcohol use in a community sample of adults [8].

Students who were offered access to the CYDU program could either agree (in which case they were immediately redirected to the site), or decline (in which case they were not redirected). Six weeks following the screening questionnaire, participants in both experimental conditions received an email link with a request to complete a follow-up survey of alcohol use. Follow-up drinking rates were evaluated to examine the potential impact of providing students with open access to web-based self-help materials on subsequent drinking behavior. Results pertaining to the intervention component of the study are reported elsewhere [5]. Upon each instance of survey access, the web-based data collection software recorded information on the web browser type used by participants to complete the survey. Of interest for the current analyses was the proportion of individuals who used a mobile device to access the survey screening survey, as well as associations of mobile device use with survey completion rates, and retention in the study during the 6-week follow-up. Both the baseline and follow-up surveys, as well as the CYDU can be viewed on a mobile devise. However, the surveys and CYDU have not been optimized for viewing on a mobile devise platform.

### Ethics and consent procedure

The study was approved by the standing research ethics board of the Centre for Addiction and Mental Health (#152/2011) and is in compliance with the Helsinki declaration for research involving human participants. Informed consent was obtained using an online consent form that the participant had to actively agree to and all participants were over the age of 16.

## Results

### Survey completion rates

Of 10,000 email invitations, 1768 students (17.7%) responded by accessing the survey link. Of these participants, 89% (1575) provided informed consent and accessed the screener questions. The vast majority (98.4%) of those who accessed the screener questions completed and submitted the survey. Attesting to the brevity of the survey, 92.8% of survey completers submitted the completed survey within 1 minute of initial access.

### Use of mobile devices for survey access

Web browser signatures collected during survey submissions were used to identify instances where survey access occurred via mobile device web browsers (e.g., iPhone, iPad, Android, Blackberry), versus traditional web browsers. Of the 1768 participants who accessed the survey, 514 (29.1% of responders) did so using mobile devices. The most common mobile device type used was the iPhone (reflecting 55% of submissions from mobile devices). Mobile phones reflected the majority of mobile devices, although a proportion used tablet software (iPad, 9% of mobile device responses). Note that the analyses were also re-conducted with the iPad coded as a traditional web browser. However, there was no difference in the pattern of results. Unfortunately, there were not enough participants using iPads to allow for a separate category.

Primary analyses examined participation outcomes as a function of whether participants accessed the surveys using a mobile device. Of participants who accessed the survey and proceeded to the consent form, mobile device users were significantly less likely than traditional users to complete the form by indicating their agreement to participate (83.1% vs. 91.9% respectively; χ^*2*^ (1) = 30.20, *p* < .001). By extension, mobile phone users were also significantly less likely to complete the entire survey (80.4% vs. 90.7% respectively; χ^*2*^(1) = 35.12, *p* < .001). Although the vast majority of those providing informed consent completed the survey, mobile device users were nonetheless more likely than non-users to terminate the survey before completion (3.3% of users vs. 1.0% of non-users; χ^*2*^(1) = 10.73, *p* < .005). An examination of survey completion time, conducted after truncating response time at 9 minutes (to account for browsers that were left open for extended periods following the initial access, which excluded 52 participants from the analysis), revealed that mobile device users took marginally longer (1.23 minutes, SD = .95) than non-users (1.05 minutes, SD = .36) to complete the survey; this difference was statistically significant (F (1) = 29.16, *p* < .001). Most participants logged onto the baseline survey within the first hour of the email invitation being sent out (80%, 1421). Of participants who responded in the first 72 hours (n = 1673), mobile device users logged on significantly faster on average (M = 484 minutes, SD = 706 minutes) than non-mobile users (M = 651 minutes, SD = 873 minutes), *F* (1, 1671) = 14.31, *p* < .001.

### Follow-up survey access rates

A total of 425 participants who met heavy drinking criteria agreed to be contacted for the 6-week follow-up survey. Of these, 29.6% had used a mobile device to access the baseline survey. A total of 294 heavy drinkers completed the follow-up survey at 6 weeks (69% of those who agreed to the 6-week follow-up). Notably, those who used a mobile device to complete the baseline screening questionnaire were significantly less likely to complete the 6-week follow-up survey (57.9% follow-up completion rate) compared to those who used a traditional operating system (73.9% follow-up completion rate), χ^*2*^(1) = 10.61, *p* = .002.

### Rates of agreement to view the web-based intervention

Of participants in the intervention condition (n = 211), 62% agreed to be sent to a website that would let them see how their drinking compares with other university students. There was no significant difference (*p* > .05) between those who used a mobile device (57.1% agreed) and those who used a traditional operating system (64.9%) on agreement to be redirected to the website. Participants who completed the 6-week follow-up (n = 294) and were in the intervention condition (n = 151) were asked if they had tried the CYDU website. There were no significant differences (*p* > .05) between those who used a mobile device (17.6%) and those who used a traditional operating system (26.5%) on stating that they had tried the website.

## Discussion

Using data from a randomized controlled trial evaluating an online alcohol intervention conducted with a sizable college student sample, we examined the impact of using mobile devices when making initial contact with a web-based intervention. This method of recruitment is distinct from another growing line of research – the use of mobile devices to collect momentary ecological response data over a period of time [9]. The current analysis was concerned with the impact of mobile devices on the completion of web-based surveys optimized for a traditional web browser (i.e., on a desktop or laptop computer). There were two hypotheses explored – that mobile device use would lead to quicker access and that mobile device use would lead to poorer completion rates. Both of these hypotheses were supported. Participants who employed a mobile device accessed the survey an average of nearly three hours earlier, which was approximately 25% faster than those who employed a standard computer internet service. However, once they had accessed the survey, participants who employed a mobile phone were less likely to complete the survey (despite its brevity) than those who used a traditional operating system. More importantly (at least for intervention trial studies), those participants who used a mobile device to respond to the survey at baseline were more than 25% less likely to complete the six-week follow-up than participants employing a regular computer Internet service.

These results would be likely to be even more pronounced in other studies. This is because the survey employed in this trial was only five items long and most participants completed it in less than two minutes. Other online intervention trials normally include considerably longer surveys. It is possible though that those participants using a mobile device who were confronted by a longer survey would simply move to a computer to respond to the survey. Our primary concern with the limitations of a mobile device to complete this type of research trial – that it would make participants less likely to complete the intervention component of the trial (in this case an online personalized feedback intervention for problem drinkers) did not gain clear support. However, this was likely because so few participants who were provided access to the online intervention actually said they tried it so that any differences in completion rates between those using a mobile device versus a traditional device would need to be very large to reach statistical significance.

There were several limitations to these analyses. Primarily, the categorization of participants’ mobile device was rather crude and based on information available in web browser signatures. We made the assumption that iPads should be categorized with other mobile devices (e.g., iPhone, Blackberry) rather than with traditional web browsers. However, the look and feel of completing a survey optimized for a computer screen would probably be easier to respond to using an iPad than on other mobile devices. Unfortunately, there were not enough participants using an iPad in the current sample to allow for a more fine grained analysis. In addition, it would be worthwhile replicating these findings using a different study sample within a research study that employed longer surveys in order to confirm these results. Nevertheless, this study does point to a growing limitation with the email list method of participant recruitment as mobile device use becomes more common. Further, it speaks to the need to consider the different platforms that participants use to reply to the online surveys in future research studies. Based on the results of the current study, attempts to maximize participant engagement and retention in web-based intervention studies by limiting access from mobile devices may be warranted. Potential strategies could include programming surveys or intervention materials so as to preempt access via mobile device software, or restricting participant contact to those times of day when respondents are most likely to have access to a desktop computer. Alternatively, some online survey material could be optimized for completion on a mobile device and the participant could be directed to this version of the survey if the online survey collection program detects connection from a mobile device web browser.

## Conclusions

This paper explored the impact of mobile devices on the completion of web-based surveys. It was determined that while mobile device users accessed a web-based survey significantly faster than their counterparts using traditional web browsers, participants who had accessed the survey on their mobile device were less likely to complete it, as well as its subsequent follow-up. These findings highlight important limitations with web-based surveys, particularly with regard to retention and engagement of participants using mobile devices.

## Competing interests

The authors declare that they have no competing interests.

## Authors’ contributions

JC, CN, NB, and CH contributed to the design of the study. JC conducted the analysis and wrote the draft of the manuscript. All authors read and approved the final manuscript.
